# Effects of qigong exercise on post-stroke cognitive impairment: a systematic review and meta-analysis of randomized controlled trials

**DOI:** 10.3389/fneur.2026.1886327

**Published:** 2026-08-05

**Authors:** Chengning Song, Huanxin Xie, Haiyan Li, Bo Lei, Bin Luo, Weijuan Gu, Huimin Wang, Yanxian Zou

**Affiliations:** 1 Shenzhen Fuyong People’s Hospital, Shenzhen, China; 2 Shenzhen Traditional Chinese Medicine Hospital, Shenzhen, China

**Keywords:** Baduanjin, Liuzijue, meta-analysis, Montreal Cognitive Assessment, post-stroke cognitive impairment, qigong, stroke, Yijinjing

## Abstract

**Background:**

Post-stroke cognitive impairment is common and negatively affects functional recovery and quality of life. Qigong is widely used in stroke rehabilitation, but its effects on cognitive outcomes remain uncertain.

**Objectives:**

To evaluate the effects of qigong exercise on cognitive function after stroke and to explore whether effects vary across qigong types (Baduanjin, Yijinjing, Liuzijue, and Dao-yin–type exercises).

**Methods:**

We systematically searched seven databases from inception to January 2026: PubMed (87 records), Embase (112), Web of Science (106), the Cochrane Library (82), CNKI (224), Wanfang (230), and VIP (147). We included randomized controlled trials in people after stroke that compared qigong with control conditions, including the same usual rehabilitation alone, health education, sham exercise, or other non-qigong interventions. Two reviewers independently screened studies, extracted data, and assessed risk of bias. The primary outcome was cognitive function measured by the Montreal Cognitive Assessment; the Mini-Mental State Examination was a supplementary outcome. Post-intervention mean and standard deviation values were preferentially extracted, and standard deviations were derived from standard errors when needed. Meta-analyses were performed in Review Manager 5.4.1 using mean differences with 95% confidence intervals under a random-effects model. Heterogeneity was assessed using *I*^2^, with subgroup analyses by qigong type and sensitivity analyses to explore heterogeneity.

**Results:**

Sixteen RCTs were included; 14 reported MoCA and 2 reported MMSE. Qigong improved MoCA versus control (random-effects model: MD = 1.87, 95% CI 1.33–2.40; *p* < 0.00001; *I*^2^ = 67%). Benefits were significant for Baduanjin (MD = 2.19, 95% CI 1.42–2.95) and Liuzijue (MD = 1.68, 95% CI 0.82–2.53), but not for Yijinjing (MD = 1.55, 95% CI − 0.28–3.38; *p* = 0.10); between-subgroup differences were not significant (*p* = 0.63). Two RCTs reported MMSE with improvement but high heterogeneity (MD = 5.82, 95% CI 2.53–9.12; *p* = 0.0005; *I*^2^ = 89%).

**Conclusion:**

Current evidence suggests that qigong improves cognitive function after stroke, with a significant increase in MoCA scores overall. Benefits were observed for Baduanjin and Liuzijue, whereas Yijinjing showed no statistically significant improvement. Two Dao-yin trials reported improved MMSE but with substantial heterogeneity. Further well-designed clinical trials are warranted to confirm these findings and clarify effects across qigong types and outcomes.

**Systematic review registration:**

Registered in PROSPERO (CRD420261320472).

## 1 Introduction

Post-stroke cognitive impairment is one of the most stubborn barriers to recovery. Many people regain a degree of strength and mobility, yet continue to struggle with slower thinking, poor attention, forgetfulness, or difficulty planning daily tasks (1, 2). These deficits can quietly undermine rehabilitation by reducing engagement with training, complicating self-management, and limiting return to work or social roles (3). Although cognitive rehabilitation and multidisciplinary care have advanced, effective options that are accessible, sustainable, and easy to integrate into routine rehabilitation are still needed (4).

Qigong is recognized in China as a formal category of traditional exercise. In national standards and official technical specifications, qigong is generally defined as a health-preservation exercise system that integrates body regulation, breath regulation, and mind regulation into coordinated practice (5). Within this framework, “Health Qigong” refers to standardized sets of routines promoted for public health and rehabilitation, and commonly includes Baduanjin, Yijinjing, and Liuzijue, among others (6, 7). In clinical rehabilitation research after stroke, qigong interventions are typically operationalized as structured training in one of these standardized routines, or in closely related Dao-yin therapeutic exercises that share the same core elements of movement, breathing, and focused attention (8–10).

The appeal of qigong in stroke rehabilitation lies in its practicality and its combined physical and cognitive demands. Practice requires sequencing and posture control, sustained attention, and breathing regulation, all of which may be relevant to cognitive recovery (11). At the same time, different routines may engage these components to different degrees. Baduanjin is generally characterized by relatively simple, repeatable movement sequences and clear rhythm (12). Liuzijue places greater emphasis on controlled breathing with prolonged exhalation coordinated with movement (13). Yijinjing typically involves larger-range movements and higher motor complexity (14). Dao-yin–type exercises, including forms derived from classical therapeutic traditions, are often delivered as guided movement-breathing routines (10). These distinctions provide a plausible basis for variability in cognitive outcomes across qigong types.

Over the past decade, several randomized controlled trials have evaluated qigong programs after stroke, most often reporting global cognition using the Montreal Cognitive Assessment and the Mini-Mental State Examination (8). However, individual trials are often small and heterogeneous. Training prescriptions vary in duration and frequency, qigong is sometimes delivered alone and sometimes as an add-on to usual rehabilitation or other therapies, and control conditions range from usual care to health education or alternative activities. This variation makes it difficult to rely on single studies to guide clinical decisions and raises a practical question: does qigong improve cognition after stroke, and are effects consistent across different routines such as Baduanjin, Liuzijue, Yijinjing, and Dao-yin–type interventions?

Existing reviews have not fully resolved this question. Some syntheses have pooled qigong together with broader exercise categories, while others have not separated qigong routines, limiting clinical interpretability. Differences in reporting formats and incomplete descriptions of allocation procedures and blinding further complicate evidence synthesis. An updated systematic review that focuses specifically on qigong, clearly defines included routines based on national and standardized practice, and examines routine type as a potential source of heterogeneity is therefore warranted.

Although several studies have investigated qigong-based interventions for cognitive outcomes after stroke, the evidence remains fragmented and has not been synthesized in a sufficiently rigorous and focused manner. Much of the available evidence on this specific topic comes from small randomized trials, Chinese-language studies, and postgraduate theses, while peer-reviewed international systematic reviews focusing specifically on qigong for post-stroke cognitive impairment remain limited. Moreover, previous evidence syntheses have often grouped qigong with broader traditional exercise or rehabilitation interventions, or have not distinguished between different qigong routines, which limits the clinical interpretability of their findings. Therefore, a focused and methodologically transparent systematic review and meta-analysis is needed to summarize the current randomized evidence, clarify the effects of different qigong routines, and provide a more accurate basis for future clinical research and rehabilitation practice.

## 2 Materials and methods

### 2.1 Search strategy

We conducted a comprehensive search of seven electronic databases, including PubMed, Embase, Web of Science, the Cochrane Library, CNKI, Wanfang, and VIP, from inception to January 2026 in accordance with the PRISMA statement to ensure a transparent and rigorous review process. The search strategy was developed using the PICOS framework, including: people with stroke and cognitive impairment (P); qigong exercise interventions including Baduanjin, Yijinjing, Liuzijue, and Dao-yin–type exercises (I); control conditions consisting of usual rehabilitation alone, health education, sham exercise, or other non-qigong interventions, with co-interventions matched whenever applicable (C); cognitive outcomes assessed using MoCA and/or MMSE, with MoCA prespecified as the primary measure and MMSE as a supplementary measure when reported(O); and randomized controlled trials (S). Search terms were constructed using a combination of controlled vocabulary and free-text keywords related to stroke, cognitive impairment, and qigong, including the names of the qigong routines. Reference lists of included studies and relevant reviews were also hand-searched to identify additional eligible trials.

### 2.2 Inclusion criteria

Studies were eligible if they met the following criteria: (1) Population: adults diagnosed with ischemic or hemorrhagic stroke with cognitive impairment; (2) Design: randomized controlled trials with a parallel-group design; (3) Intervention: qigong exercise interventions including Baduanjin, Yijinjing, Liuzijue, and Dao-yin–type exercises, delivered alone or in combination with usual rehabilitation; (4) Comparator: usual rehabilitation alone, health education, sham exercise, or other non-qigong interventions, with co-interventions matched between groups when applicable; and (5) Outcomes: reporting cognitive function assessed at baseline and post-intervention using the MoCA and/or the MMSE, with sufficient data to extract or calculate effect estimates. MoCA was prespecified as the primary cognitive outcome, and MMSE was considered a supplementary outcome when reported.

### 2.3 Exclusion criteria

Studies were excluded if they met any of the following criteria: (1) non-randomized designs such as observational studies, quasi-experiments, case series or case reports, reviews, protocols, and conference abstracts without full data; (2) participants were not stroke patients with cognitive impairment, or outcomes were not specific to post-stroke cognitive function; (3) the intervention did not include qigong, or the qigong program was unclear or insufficiently described to determine that it involved Baduanjin, Yijinjing, Liuzijue, or Dao-yin–type exercises; (4) interventions primarily based on other exercise modalities rather than qigong, such as aerobic training, resistance training, Tai Chi, yoga, Pilates, or dance, unless the effects of qigong could be isolated; (5) co-interventions differed between groups such that the effect of adding qigong could not be separated; and (6) did not report either the MoCA or the MMSE, or lacked sufficient post-intervention data for extraction or calculation of effect estimates.

### 2.4 Study selection

Literature screening and study selection were managed in EndNote. Two reviewers (Song CN and Xie HX) independently screened titles and abstracts, removed duplicates, and excluded records that were clearly irrelevant to the review question. Records that did not meet the eligibility criteria were excluded at this stage, including those with ineligible study designs or insufficient information for assessment. The same reviewers then retrieved and independently assessed full texts for eligibility, documenting reasons for exclusion. Disagreements were resolved through discussion; when consensus could not be reached, a third reviewer (Zou YX) adjudicated. The final list of included studies was cross-checked by Feng NN for completeness and consistency.

### 2.5 Data extraction

A standardized data extraction form was developed and piloted before formal data collection. Two reviewers (Song CN and Xie HX) independently extracted data from all included studies. Any discrepancies were resolved through discussion; if consensus could not be reached, a third reviewer (Zou YX) adjudicated. For each trial, we extracted author and publication year, participant characteristics, intervention and control protocols, sample size for each group, cognitive outcome measures and assessment time points, and outcome data required for meta-analysis. Training dose parameters were also extracted, including session duration, frequency, intervention period, and total practice time. Extracted data were cross-checked by Luo B for completeness and consistency. When outcomes were reported as mean and standard error, standard deviations were calculated by Xie HX and verified by Song CN according to standard formulas.

### 2.6 Risk of bias of individual studies

Two reviewers (Lei B and Haiyan L) independently assessed risk of bias using the Cochrane risk-of-bias tool according to the Cochrane Handbook (version 5.1.0). Seven domains were evaluated: random sequence generation, allocation concealment, blinding of participants and personnel, blinding of outcome assessment, incomplete outcome data, selective reporting, and other bias. Each domain was judged as low, unclear, or high risk of bias. Disagreements were resolved through discussion; if consensus could not be reached, a third reviewer (Gu WJ) arbitrated. Risk-of-bias tables and summary figures were generated and checked by Wang HM for consistency.

### 2.7 Data analysis

Meta-analyses were conducted in Review Manager 5.4.1. Continuous outcomes were pooled as mean differences with 95% confidence intervals. A random-effects model was specified *a priori* given anticipated clinical and methodological heterogeneity across trials, including differences in qigong type, training dose, co-interventions, and control conditions. Statistical heterogeneity was assessed using the *I*^2^ statistic. Prespecified subgroup analyses were performed by qigong type, including Baduanjin, Yijinjing, Liuzijue, and Dao-yin–type exercises. When multiple post-intervention time points were reported, end-of-treatment values were preferentially used for the primary analysis. Sensitivity analyses were performed by omitting one study at a time and by excluding influential studies to assess the robustness of pooled estimates. When data were incomplete or not directly extractable, we attempted to contact corresponding authors; when unavailable, summary statistics were derived from reported information using methods recommended in the Cochrane Handbook.

## 3 Results

### 3.1 Study identification and selection

**Figure 1:**
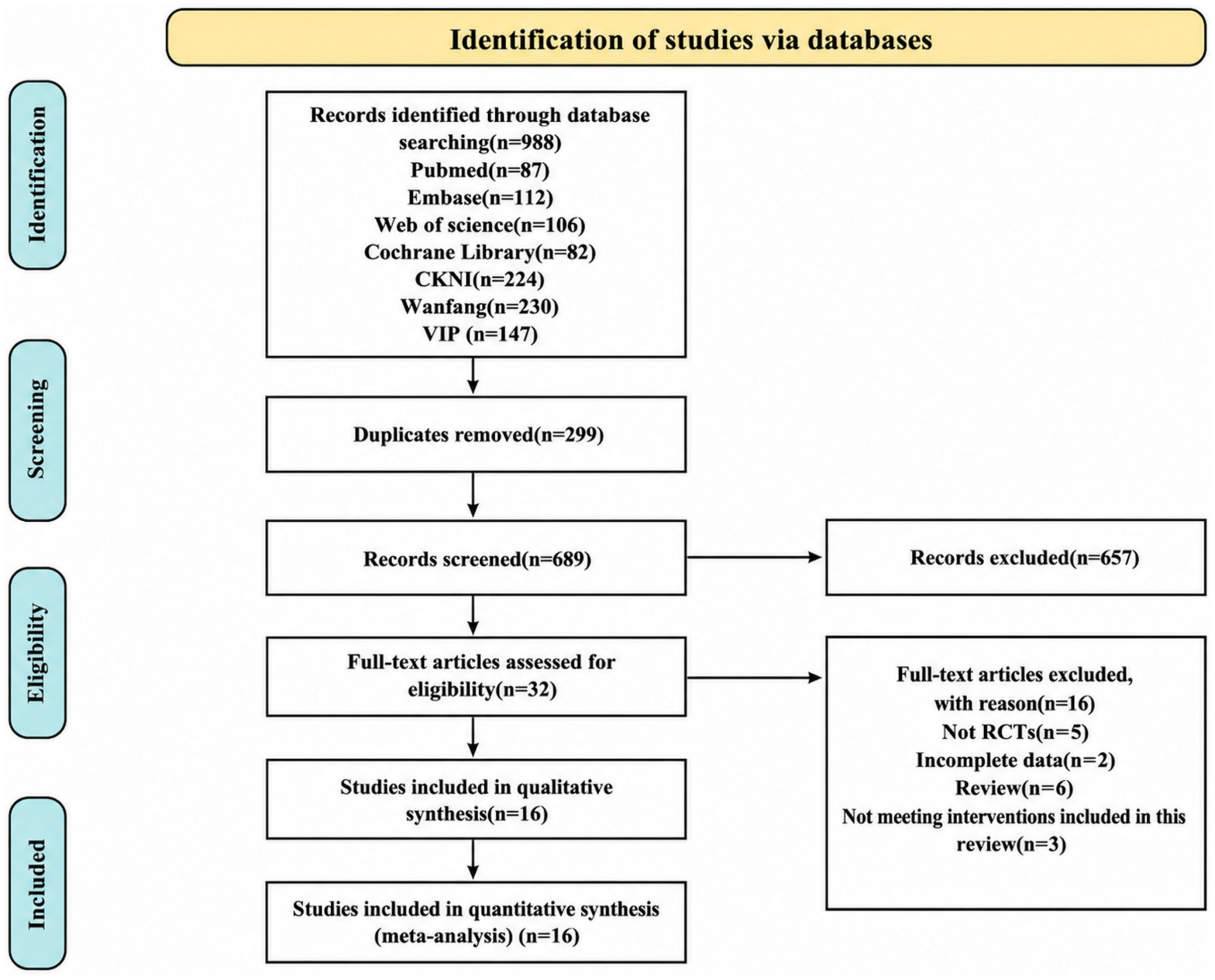
PRISMA flow diagram of study identification, screening, eligibility assessment, and inclusion.

A total of 988 records were identified through database searching, including PubMed (87), Embase (112), Web of Science (106), the Cochrane Library (82), CNKI (224), Wanfang (230), and VIP (147). After removing 299 duplicates, 689 unique records remained for title and abstract screening, of which 657 were excluded. 32 full-text articles were assessed for eligibility, and 16 were excluded with reasons. Finally, 16 studies were included in the qualitative synthesis and quantitative synthesis. Fourteen studies reporting MoCA were included in the primary meta-analysis, and two studies reporting MMSE with Dao-yin interventions were included as a supplementary analysis. The study selection process is shown in Figure 1.

### 3.2 Study characteristics

Sixteen RCTs were included. The trials enrolled adults with ischemic or hemorrhagic stroke and cognitive impairment, spanning subacute to chronic stages. Interventions consisted of qigong exercise programs, including Baduanjin, Yijinjing, Liuzijue, and Dao-yin, delivered alone or as an adjunct to usual rehabilitation. Control conditions typically involved usual rehabilitation alone, health education, sham exercise, or other non-qigong interventions. Training prescriptions varied across studies in frequency and duration. Fourteen trials reported MoCA at baseline and at end of treatment, and two trials reported MMSE. Detailed study characteristics, intervention protocols, and assessment time points are summarized in Table 1.

**Table 1:** Characteristics of included randomized controlled trials and qigong training protocols.

Study	Treatment group				Control group				Treatment duration	Total minutes	Qigong type	Assessment tool
	Intervention	*N*	Mean	SD	Intervention	*N*	Mean	SD				
Zheng (2020) (21)	Baduanjin plus rehabilitation therapy	22	24.83	2.44	Rehabilitation	19	22.29	2.35	40 min × 3/wk × 24 wk	2,880	Baduanjin	MoCA
Feng (2022) (22)	Baduanjin plus acupuncture and rehabilitation therapy	47	26.95	1.83	Rehabilitation	47	25.61	1.97	40 min × 5/wk × 8 wk	1,600	Baduanjin	MoCA
Zheng (2018) (23)	Baduanjin	22	25.27	2.31	Health education	19	22.11	3.43	40 min × 3/wk × 24 wk	2,880	Baduanjin	MoCA
Xiong (2018) (24)	Baduanjin	22	25.05	2.28	Health education	19	22.84	2.09	40 min × 3/wk × 24 wk	2,880	Baduanjin	MoCA
Yu (2022) (25)	Baduanjin plus acupuncture	30	24. 31	4. 05	Acupuncture	30	21.15	4.16	20–40 min × 7/wk × 4 wk	1,520	Baduanjin	MoCA
Jin (2021) (26)	Modified Yijinjing plus rehabilitation therapy	27	21.26	2.96	Rehabilitation	24	21.79	4.88	40 min × 5/wk × 4 wk	800	Yijinjing	MoCA
Xue (2020) (27)	Modified Yijinjing plus rehabilitation therapy	24	24.92	1.67	Rehabilitation	22	22.83	2.22	40 min × 5/wk × 4 wk	800	Yijinjing	MoCA
Zhang (2019) (28)	Modified Yijinjing plus rehabilitation therapy	27	18.7	5.04	Rehabilitation	29	15.66	5.07	60 min × 3/wk × 4 wk	720	Yijinjing	MoCA
Qi (2022) (29)	Dao-yin plus rehabilitation therapy	30	28.73	3.65	Rehabilitation	30	24.71	4.18	40 min × 6/wk × 2 wk	480	Dao-yin	MMSE
Lin (2022) (30)	Dao-yin plus rehabilitation therapy	60	27.85	2.88	Rehabilitation	60	20.46	2.37	40 min × 6/wk × 2 wk	480	Dao-yin	MMSE
Yu (2021) (31)	Liuzijue plus rehabilitation therapy	16	23.56	1.71	Rehabilitation	16	22.31	1.66	24 min × 7/wk × 12 wk	2016	Liuzijue	MoCA
Song (2025) (32)	Liuzijue plus acupuncture and rehabilitation therapy	40	27.33	3.70	Acupuncture plus rehabilitation	40	25.33	2.22	24 min × 5/wk × 6 wk	720	Liuzijue	MoCA
Ren (2025) (33)	Liuzijue plus acupuncture and rehabilitation therapy	59	28.72	1.48	Acupuncture plus rehabilitation	59	25.92	1.67	30 min × 5/wk × 12 wk	1800	Liuzijue	MoCA
Zhuang (2025) (34)	Liuzijue plus respiratory training and executive function training	36	24.34	1.02	Respiratory training plus executive function training	36	23.70	1.54	30 min × 5/wk × 8 wk	1,200	Liuzijue	MoCA
Liu (2022) (35)	Liuzijue plus rehabilitation therapy and nursing care	17	23.56	1.71	Rehabilitation plus nursing care	17	22.31	1.66	NR	NR	Liuzijue	MoCA
Liu (2023) (36)	Liuzijue plus rehabilitation therapy	30	24.92	1.67	Rehabilitation	30	22.83	2.22	NR	NR	Liuzijue	MoCA

(1) MoCA, Montreal Cognitive Assessment; MMSE, Mini-Mental State Examination; SD, standard deviation; SE, standard error; NR, not reported. (2) Outcome data: Values are presented as mean ± SD at end of treatment unless otherwise stated. (3) SE conversion: When studies reported mean ± SE, SD was calculated as SD = SE × √n. (4) Training dose: Total practice time (min) was calculated as minutes per session × sessions per week × total weeks. (5) Qigong types: Baduanjin, Yijinjing, Liuzijue, and Dao-yin.

### 3.3 Quality assessment of the included studies

**Figure 2:**
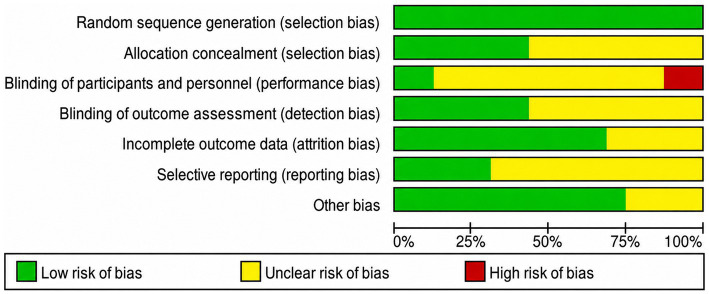
Risk of bias graph of included studies in this systematic review.

**Figure 3:**
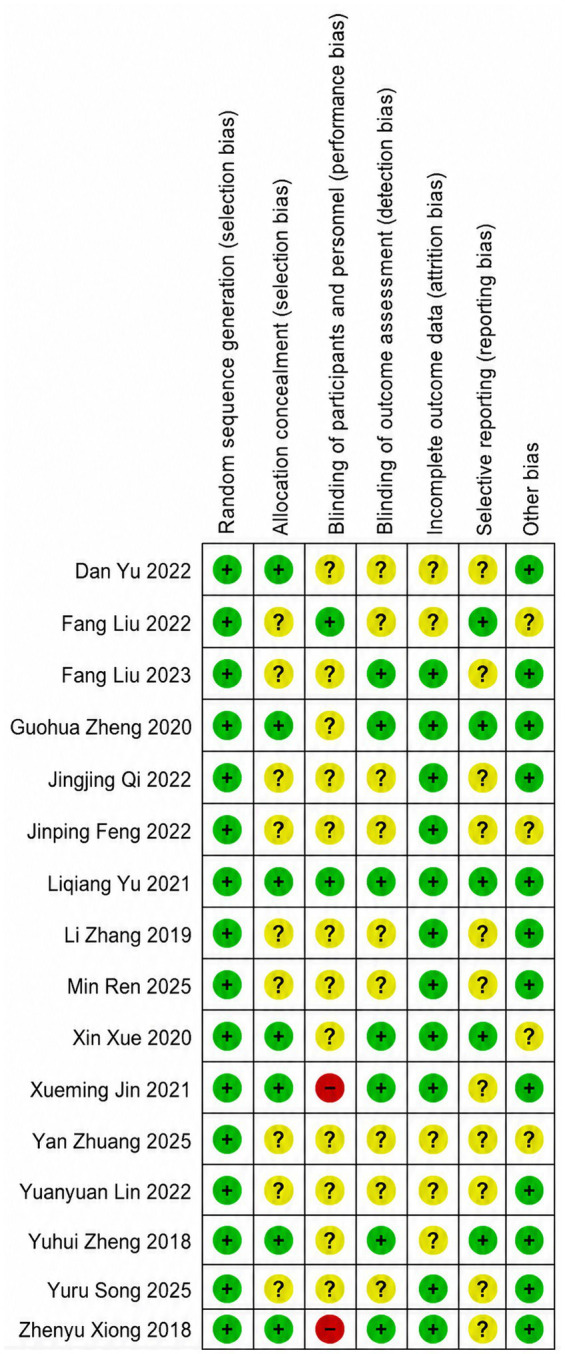
Risk of bias summary of included studies in this systematic review.

Risk of bias was assessed using the Cochrane RoB 1.0 tool, and the findings are shown in Figures 2, 3. Overall, the included studies showed a mixed risk-of-bias profile. All 16 trials were rated as low risk for random sequence generation because they reported the use of random allocation methods, such as random number tables or computer-generated random sequences. Allocation concealment was rated as low risk in 6 trials and unclear risk in 10 trials; the unclear judgments were mainly due to insufficient reporting of concealment procedures, such as sealed envelopes, independent allocation, or central randomization. For blinding of participants and personnel, 2 trials were rated as low risk, 12 as unclear risk, and 2 as high risk. This domain represented the main methodological concern, as qigong is a behavioral exercise intervention and participant/personnel blinding is difficult or not feasible when sham exercise or attention-matched control conditions are not used. Blinding of outcome assessment was rated as low risk in 6 trials and unclear risk in 10 trials; unclear judgments were assigned when assessor blinding was not explicitly described. Incomplete outcome data were rated as low risk in 11 trials and unclear risk in 5 trials, mainly depending on whether attrition, dropout reasons, and missing data handling were clearly reported. Selective reporting was rated as low risk in 5 trials and unclear risk in 11 trials, largely because trial registration or published protocols were unavailable in several studies, making it difficult to verify whether all prespecified outcomes were reported. Other bias was rated as low risk in 12 trials and unclear risk in 4 trials; unclear judgments mainly reflected potential concerns such as small sample sizes, insufficient details regarding co-interventions, or possible imbalance in intervention exposure. Overall, the main methodological concerns were related to allocation concealment, blinding of participants and personnel, assessor blinding, and selective reporting.

### 3.4 Interventions

Across the included trials, the intervention arms received structured qigong training delivered as a standardized routine, either as a stand-alone program or as an add-on to usual rehabilitation. Four qigong modalities were used: Baduanjin, Yijinjing, Liuzijue, and Dao-yin. Baduanjin was commonly implemented as supervised group practice and in several studies was combined with usual rehabilitation, while a smaller number of trials added acupuncture as a co-intervention. Yijinjing interventions were typically reported as modified routines and were delivered alongside rehabilitation programs. Liuzijue was administered as guided breathing–movement training and was most often combined with rehabilitation; in some studies it was delivered together with acupuncture or additional training components. Dao-yin interventions in the included studies were based on Chaoyuanfang Dao-yin and were provided in combination with usual rehabilitation.

Training prescriptions varied across trials. Session duration ranged from 20 to 60 min, with most studies using 40-min sessions. Practice frequency ranged from three to seven sessions per week, and total intervention length ranged from 2 to 24 weeks. Using the reported dose parameters, total practice time varied widely across studies, from several 100 min to more than 2000 min over the full intervention period. Most trials delivered qigong under supervision during the intervention phase, and control groups generally received usual rehabilitation, health education, or matched co-interventions without qigong.

### 3.5 Comparator interventions

Comparator conditions varied across trials but were designed to reflect routine care or non-qigong alternatives. Most studies used usual rehabilitation as the control, typically comprising conventional rehabilitation therapies and training delivered at the same treatment setting and duration as the intervention group, without qigong practice. Several trials used health education as the comparator, providing routine counseling and stroke-related guidance without structured exercise training. In studies where the intervention combined qigong with another therapy, the comparator generally received the same co-intervention to isolate the added effect of qigong. Specifically, trials that added acupuncture in the qigong arm used acupuncture alone or acupuncture plus usual rehabilitation in the control arm, and one trial matched additional conventional respiratory training and executive function training in both groups while withholding qigong in the control arm.

### 3.6 Main outcomes

**Figure 4:**
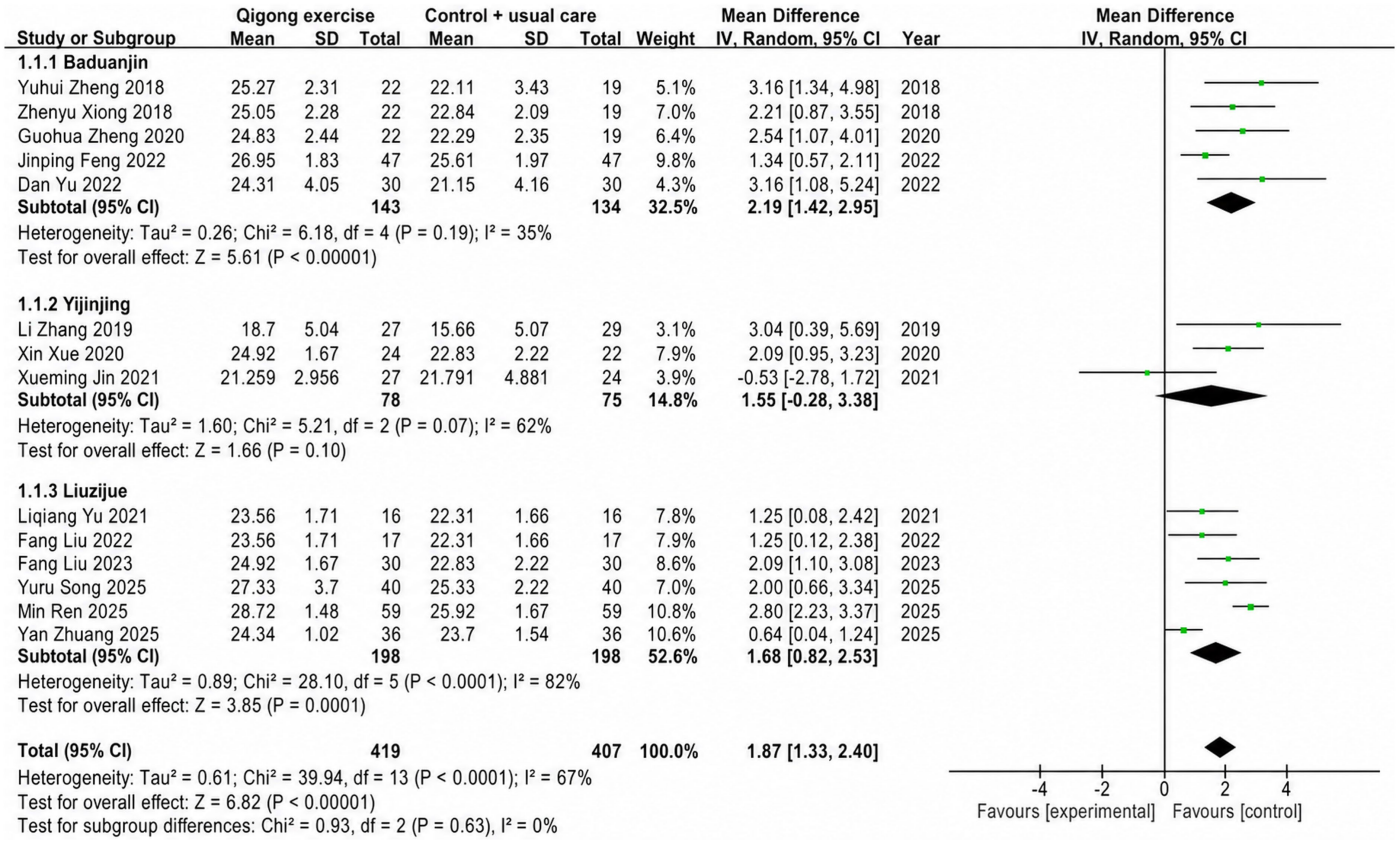
Forest plot of MoCA comparing qigong exercise versus control in post-stroke cognitive impairment.

Figure 4 presents the pooled effects of qigong on cognitive function measured by MoCA. Fourteen trials were included in the meta-analysis. Overall, qigong significantly improved MoCA scores compared with control under a random-effects model, with a mean difference of 1.87 points and a 95% confidence interval from 1.33 to 2.40. Statistical heterogeneity was moderate to substantial, with an *I*^2^ of 67%. Subgroup analyses by qigong type showed significant improvements for Baduanjin, with a mean difference of 2.19 and a 95% confidence interval from 1.42 to 2.95, and for Liuzijue, with a mean difference of 1.68 and a 95% confidence interval from 0.82 to 2.53. In contrast, Yijinjing did not show a statistically significant effect, with a mean difference of 1.55 and a 95% confidence interval from −0.28 to 3.38. The test for subgroup differences was not significant, indicating no evidence that effects differed across qigong types.

Two trials reported MMSE outcomes using Dao-yin interventions. Meta-analysis showed higher MMSE scores in the qigong group than in controls under a random-effects model, with a mean difference of 5.82 and a 95% confidence interval from 2.53 to 9.12, but heterogeneity was substantial with an *I*^2^ of 89%.

### 3.7 Sensitivity analysis

Sensitivity analyses were performed to assess the robustness of pooled effects and to investigate heterogeneity. For the primary MoCA analysis, omitting studies one at a time did not change the overall direction of effect, indicating that the main finding was not driven by any single trial. Heterogeneity was most prominent in the Liuzijue subgroup, so influence analyses were conducted to identify potential contributors. Excluding Zhuang 2025 reduced *I*^2^ from 82 to 58%, and excluding Ren 2025 reduced *I*^2^ to 49%; removing both studies reduced *I*^2^ to 0%, suggesting that heterogeneity in this subgroup was largely driven by these two trials. The observed variability was plausibly related to differences in intervention composition and study methods, including multi-component training added to Liuzijue in some trials, variation in co-interventions such as acupuncture or additional rehabilitation elements, differences in training dose and treatment duration. For MMSE, only two Dao-yin trials were available and heterogeneity remained substantial with *I*^2^ of 89%. Switching between random-effects and fixed-effect models yielded consistent statistical significance but did not meaningfully reduce heterogeneity, indicating that the between-study variability was unlikely to be explained by the choice of pooling model and may reflect differences in participant characteristics or usual rehabilitation implementation.

## 4 Discussion

This systematic review and meta-analysis synthesized evidence from randomized trials evaluating qigong for post-stroke cognitive impairment. The main finding was that qigong was associated with a statistically significant improvement in global cognition measured by MoCA, with an average increase of about two points compared with control. This magnitude is meaningful in the context of post-stroke rehabilitation, where modest gains in global cognition can translate into better engagement with therapy, improved self-management, and greater independence in daily activities. Subgroup analyses did not demonstrate a significant difference across qigong types, suggesting that, at the level of currently available evidence, qigong as a class of intervention has a consistent direction of benefit. At the same time, the strength and consistency of evidence varied by routine. Baduanjin showed a clear and relatively stable effect with lower heterogeneity, Liuzijue also showed benefit but with greater variability, and Yijinjing showed only a non-significant trend, indicating that evidence for this routine remains less conclusive.

The observed cognitive benefits may also be supported by several plausible biological pathways. As a mind–body exercise, qigong integrates slow movement, postural control, breathing regulation, and focused attention, which may jointly stimulate motor-cognitive integration and activity-dependent neuroplasticity (5, 15). At the molecular level, exercise-related neurotrophic modulation may be one potential pathway linking qigong practice with cognitive improvement. Previous human studies have suggested that meditation and mind–body exercise may influence circulating brain-derived neurotrophic factor (BDNF) levels, and similar traditional mind–body interventions have also been investigated for their effects on cognitive-related biomarkers. As BDNF is a key mediator of synaptic plasticity, learning, and memory, exercise-induced changes in BDNF may provide a plausible biological basis for the cognitive benefits observed after qigong training (16, 17). In addition, qigong-related slow movement and breathing regulation may help reduce oxidative stress and inflammatory activation, both of which are involved in post-stroke neuronal injury and cognitive decline (18). Evidence from Tai Chi and Qigong studies suggests that these practices may improve oxidative stress balance and modulate inflammatory profiles, including antioxidant enzymes, malondialdehyde, interleukin-6, tumor necrosis factor-*α*, and C-reactive protein (19). These mechanisms may provide a biological basis for the improvements in cognitive scores observed in the present review. However, because most included trials did not directly measure these biomarkers, these pathways should be interpreted as plausible explanations rather than confirmed mediators. Future trials should incorporate biomarkers such as BDNF, oxidative stress markers, and inflammatory cytokines to clarify the biological mechanisms linking qigong practice with cognitive recovery after stroke.

From a practical perspective, intervention duration and total exercise dose should be considered when applying qigong programs in stroke rehabilitation. Among the 14 studies that clearly reported training dose, the intervention duration ranged from 2 to 24 weeks, with an average duration of approximately 9.9 weeks. Training frequency ranged from 3 to 7 sessions per week, and most sessions lasted 24–60 min. The total reported exercise dose ranged from 480 to 2,880 min, with an average of approximately 1,484 min, equivalent to about 24.7 h. These findings suggest that qigong interventions in the current evidence base generally require repeated practice over several weeks rather than short-term exposure. However, because the included trials differed in qigong type, training intensity, comparator, and outcome assessment time points, the current evidence is insufficient to determine a definitive minimum effective number of training hours. Future trials should directly compare different intervention durations and total training doses to establish more precise, evidence-based qigong prescriptions for post-stroke cognitive impairment.

Heterogeneity is an important consideration when interpreting pooled estimates. The overall MoCA analysis showed moderate to substantial heterogeneity, and heterogeneity was most pronounced in the Liuzijue subgroup. Sensitivity analyses provided a clear signal that variability in the Liuzijue subgroup was largely driven by a small number of studies. Removing Zhuang 2025 or Ren 2025 substantially reduced heterogeneity, and excluding both eliminated it. This pattern suggests that the direction of effect is stable but that certain trials differed in ways that materially affected effect size. Several sources of clinical and methodological variation may explain this. First, some Liuzijue trials implemented multi-component programs by adding cognitive or executive-function training elements, which makes the intervention more intensive than Liuzijue practice alone and can inflate benefits beyond the contribution of qigong. Second, co-interventions such as acupuncture or electroacupuncture were not uniform across trials; even when both groups received a co-intervention, differences in implementation detail may persist. Third, training dose varied substantially across studies in session duration, weekly frequency, and total weeks, and dose differences are likely to influence cognitive outcomes. Finally, study design features, including incomplete reporting of allocation concealment and assessor blinding, may have introduced bias and contributed to between-study variability. Together, these factors support interpreting subgroup findings with attention to intervention composition rather than treating all Liuzijue trials as equivalent.

The MMSE was reported in only two Dao-yin trials and showed improvement in pooled analysis; however, heterogeneity was very high and remained high after switching between fixed-effect and random-effects models. With only two studies, heterogeneity estimates are inherently unstable, and model choice cannot resolve true clinical differences. The persistence of heterogeneity therefore likely reflects differences in participant characteristics, baseline cognitive status, or the content and intensity of usual rehabilitation delivered alongside Dao-yin. In addition, MMSE is less sensitive to certain cognitive domains and may be more susceptible to ceiling effects, particularly in milder impairment, which can further complicate comparisons across small trials. For these reasons, MMSE findings should be viewed as supportive but preliminary, while MoCA appears to provide a more robust and informative primary outcome for future qigong trials in post-stroke populations.

This review has limitations that should temper interpretation and guide future research. Many trials were small and several domains of risk of bias were rated as unclear because key methodological details were not fully reported, particularly allocation concealment and blinding. Intervention prescriptions were heterogeneous, and adherence monitoring was inconsistently described, limiting inferences about dose–response relationships. Control conditions varied from usual rehabilitation to health education and other active comparators, which affects the expected incremental benefit of qigong. Another important limitation is that we could not perform reliable subgroup analyses according to stroke type or time since stroke onset. Ischemic and hemorrhagic stroke may differ in lesion characteristics, recovery trajectories, and mechanisms of cognitive impairment. Similarly, patients in the subacute and chronic stages may show different levels of spontaneous recovery, neuroplastic potential, and responsiveness to rehabilitation interventions (1, 20). These factors could influence the magnitude of cognitive improvement observed after qigong training. However, the included trials did not consistently report stroke subtype, lesion location, disease duration, or onset stage in sufficient detail, which prevented further stratified analyses. Future trials should clearly report stroke type and time since onset, and future evidence syntheses should examine whether these clinical characteristics modify the effects of qigong on post-stroke cognitive recovery. Follow-up assessments were uncommon, so the durability of cognitive gains remains uncertain. Evidence for Yijinjing was limited and did not demonstrate a statistically significant MoCA improvement, highlighting the need for additional well-designed trials focused on this routine. Future RCTs should pre-register protocols, apply rigorous randomization and concealment procedures, standardize reporting of training dose and adherence, define co-interventions clearly, and prioritize consistent cognitive endpoints with prespecified time points, including longer-term follow-up. Such improvements would strengthen the evidence base and help identify the most effective qigong prescriptions for cognitive recovery after stroke.

## 5 Conclusion

Current evidence from randomized trials suggests that qigong improves cognitive function after stroke, with a significant increase in MoCA scores compared with control. Benefits were evident for Baduanjin and Liuzijue, whereas Yijinjing showed only a non-significant trend. Evidence based on MMSE was limited to two Dao-yin trials and showed improvement but with substantial heterogeneity, so it should be interpreted cautiously. Overall, qigong appears to be a promising adjunct to post-stroke rehabilitation for cognitive recovery, and further well-designed trials with standardized protocols, clear co-interventions, and longer follow-up are needed to confirm effects and clarify optimal routines and training dose.

## Data Availability

The original contributions presented in the study are included in the article/supplementary material, further inquiries can be directed to the corresponding author.
